# A Machine Learning Approach Reveals Distinct Predictors of Vaping Dependence for Adolescent Daily and Non-Daily Vapers in the COVID-19 Era

**DOI:** 10.3390/healthcare11101465

**Published:** 2023-05-18

**Authors:** Ishmeet Singh, Varna Valavil Punnapuzha, Nicholas Mitsakakis, Rui Fu, Michael Chaiton

**Affiliations:** 1 Institute for Mental Health Policy Research, Centre for Addiction and Mental Health, Toronto, ON M5S 2S1, Canada; 2 Dalla Lana School of Public Health, University of Toronto, Toronto, ON M5T 3M7, Canadarui.fu@mail.utoronto.ca (R.F.); 3 Children’s Hospital of Eastern Ontario Research Institute, Ottawa, ON K1H 8L1, Canada; 4 Department of Otolaryngology—Head and Neck Surgery, Sunnybrook Research Institute, University of Toronto, Toronto, ON M4N 3M5, Canada

**Keywords:** electronic cigarettes, vaping dependence, machine learning, lasso regression

## Abstract

Since 2016, there has been a substantial rise in e-cigarette (vaping) dependence among young people. In this prospective cohort study, we aimed to identify the different predictors of vaping dependence over 3 months among adolescents who were baseline daily and non-daily vapers. We recruited ever-vaping Canadian residents aged 16–25 years on social media platforms and asked them to complete a baseline survey in November 2020. A validated vaping dependence score (0–23) summing up their responses to nine questions was calculated at the 3-month follow-up survey. Separate lasso regression models were developed to identify predictors of higher 3-month vaping dependence score among baseline daily and non-daily vapers. Of the 1172 participants, 643 (54.9%) were daily vapers with a mean age of 19.6 ± 2.6 years and 76.4% (*n* = 895) of them being female. The two models achieved adequate predictive performance. Place of last vape purchase, number of days a pod lasts, and the frequency of nicotine-containing vaping were the most important predictors for dependence among daily vapers, while race, sexual orientation and reporting treatment for heart disease were the most important predictors in non-daily vapers. These findings have implications for vaping control policies that target adolescents at different stages of vape use.

## 1. Introduction

Electronic cigarettes, known as e-cigarette or vaping, have become a significant public health concern. During the past five years, Canada has seen a modest increase in the percentage of youth and young adults who have ever tried vaping (from 21.3% to 29% and from 26.1% to 48%), while the proportion of daily vapers has risen substantially by 25% (from 44% to 55% among current past month vapers) in just 2 years (from 2019 to 2021) [1]. These concerning trends may be attributed to tobacco companies promoting electronic devices and heated tobacco as a less harmful alternative to traditional cigarettes, capitalizing on growing health concerns. Companies such as Philip Morris International are even phasing out traditional cigarette lines in favor of these new products, intending to market them as a substitute [2]. For young people, especially adolescents, there are concerns about the potential long-term health effects of these products and their potentials to serve as a gateway to traditional cigarette use. Assessing these consequences of vaping requires in-depth understanding on the addictive behaviors and the associated predictors.

Specific indices that measure the degree of self-perceived vaping dependence have been developed. These include the 10-item Penn State Electronic Cigarette Dependence Index (PS-ECDI) [3], the E-Cigarette Dependence Scale (EDS) [4,5], a three-point addiction scale [6], and total expenditures spent on buying vaping products [7]. Using these measures, high levels of vaping dependence has been linked to a number of factors, the most prominent of which are directly related to the patterns of vaping such as high nicotine concentration, vaping shortly after waking up, vaping frequently, and long vaping sessions [3,4,5,7,8]. Furthermore, social factors such as having friends or family members who vape, being frequently exposed to vaping advertisements, use of social media, and the availability of a variety of flavors and vaping devices have also been shown to increase an adolescent’s susceptibility to vaping, and subsequently, addiction to vaping [9,10,11,12,13,14]. Demographic (such as age, race, and education) and psychological factors (such as having anxiety) may also contribute to vaping dependence [14,15,16]. The place of obtaining vaping devices has emerged in recent studies as another factor that might affect risk of dependence; specifically, buying an e-cigarette using social resources such as from a family member or a friend has been found to be associated with current (past 30-day) and frequent vaping in youth [1,17].

Although these previous studies have identified some potential predictors of vaping dependence, those analyses were not stratified by daily and non-daily vapers [18,19]. The cigarette literature has suggested that the set of predictors for experimental use (primarily social and demographic factors) differed substantially from predictors for dependence (primarily product related factors); this warrants separate analyses to identify the predictors of vaping dependence by baseline vaping status. Furthermore, as we rebuild vaping prevention programs in the post-pandemic era, there is a lack of evidence from the literature that specifically identifies pandemic-related factors that lead to addiction in young people [8,20,21].

A recent study points out that environments of tobacco research are increasingly complicated and advanced analytical tools are required to tackle the vast volumes of data and specialized activities involved in eradicating tobacco-related health issues [22]. Supervised machine learning is one such technique that has been proven valuable in drug discovery, public health, and chronic disease management research [23,24,25,26]. Compared to traditional regression methods that are restrictive as they limit the volume and form of variables that can be examined as candidate predictors, supervised machine learning provides a means to skip distributional assumptions to characterize potentially meaningful associations in a flexible, data-driven, and exploratory manner [8,21]. Findings of such machine learning-guided descriptive epidemiology studies are hypothesis-generating, and could thereby provide very useful implications to guide more focused investigations in the future.

In this study, we used penalized regression, a supervised machine learning method, to predict a validated score denoting degree of self-reported vaping dependence over a course of 3 months for baseline daily and non-daily vapers by considering a broad pool of variables [27]. Specific predictors of high vaping dependence were identified for the two groups of baseline vapers to inform the development of targeted strategies. Variables that describe the perceived COVID-19 pandemic impact on substance use were considered to provide timely findings that could guide policy decision-making.

## 2. Materials and Methods

### 2.1. Study Design and Population

This prospective cohort study took place from August 2020 to February 2021, a period that roughly coincides with the first and second waves of the COVID-19 pandemic in Canada [28,29]. As part of the Ontario Tobacco Research Unit Youth and Young Adult Research Registration Panel Study [5,6], we initially conducted a convenience sampling using Instagram advertisements to recruit Canadian residents aged 16–25 years. The advertisements included a link to the screening survey directing the participants to the REDCap platform. Of the 5143 individuals who clicked on the link, passed the screening survey, and were subsequently invited to complete the baseline survey, 3082 of them responded (response rate 59.9%) and provided a written consent to participate in this study. The study was approved by the Research Ethics Board of the Centre for Addiction and Mental Health.

We administered a baseline (Wave 1) survey online in November 2020 to all participants to collect detailed data on their sociodemographic characteristics, health status, substance use, vaping behaviours, and impacts of the pandemic on their lifestyle. Participants were followed up 3 months later via emails to complete a similar online questionnaire with updated items reflecting potential progression in substance use, including vaping. In addition to entry into a draw for an additional gift card, we incentivized the participants via CAD 10 gift cards with an increasing value of prize money per each successive survey completed.

### 2.2. Study Cohort

**Figure A1:**
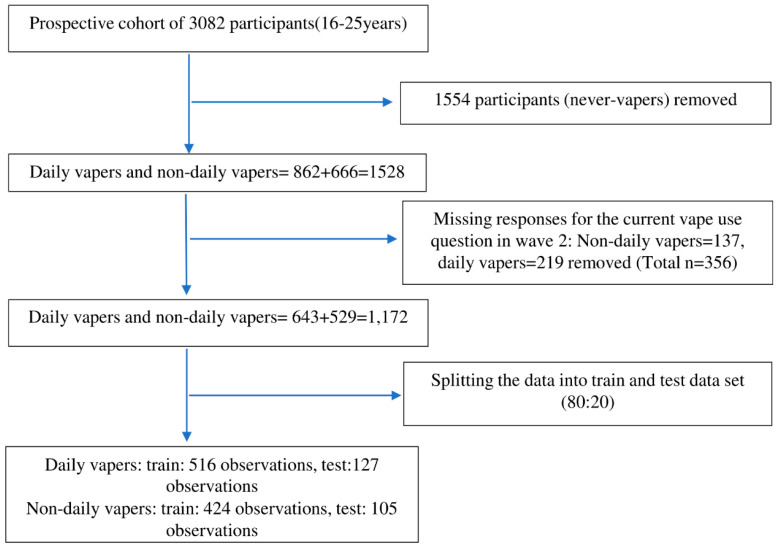
A flow diagram showing the inclusion of participants in the study cohort.

The present analysis was conducted on participants who reported to have ever tried an e-cigarette (i.e., ever-vapers) at the Wave 1 survey. We further restricted the cohort to be those who were successfully followed up 3 months later at Wave 2 for calculating a vaping dependence score, the outcome of this analysis (see below). These exclusions yielded 1172 participants in the study cohort (Appendix A, Figure A1). We conducted separate analyses for baseline ever-vapers who were daily (including almost daily) and non-daily (weekly, monthly, less than monthly) vapers.

### 2.3. Outcome

A continuous outcome variable was created to represent self-reported vaping dependence measured 3 months from the baseline. This measure, adapted from the PS-EDI has been validated on multiple occasions [3,5]. Responses to nine questions at Wave 2 on perceived urges to vape, puff numbers, and potential withdrawal symptoms were used to compute a score ranging from 0–23 where higher scores indicated increased vaping dependence (Appendix A, Table A1). Participants who did not answer any of the nine questions were excluded from the analysis; however, we chose to retain those who skipped some (but not all) of the nine questions by assigning 0 to be their vaping dependence score.

### 2.4. Potential Predictors

We considered 169 variables measured at baseline to predict the vaping dependence score 3 months later. These variables encompassed a wide range of individual-level characteristics. For sociodemographic variables, we measured age, sex, gender, province, race, marital status, sexual orientation, and education. For health and behaviour status, we recorded self-rated physical and mental health and a validated score reflecting sensation-seeking behaviours (such as frightening things, new and exciting experiences, and rule-breaking friends [6,30]). We also collected data on current use of substances, including cigarette, marijuana, hookah, shisha/waterpipe, alcohol, and other tobacco products (cigars, pipes, chewing tobacco, and bidis). Susceptibility (number of close friends who are users), perceived long-term health risks, and the influence of COVID-19 on the use of these substances were also collected [31,32]. In-depth survey questions elicited data on the behaviours, preferences, and perceptions of vaping. This included participant age when first vaped, place where one got their last vaping device, flavor of vape used on initiation and in past 3 months, use of vaping device for dripping, frequency of vaping, vaping with nicotine, nicotine concentration and nicotine salts in e-liquid, the type of e-liquid/pod/cartridge used, duration of pod lasting, the side effects from vaping (cold, cough, phlegm, wheeze or shortness of breath), and exposure to vaping advertising. Variables used to create the vaping dependence score was removed.

### 2.5. Data Pre-Processing

We prepared separate datasets for baseline daily and non-daily vapers. Missing data were infrequent for both groups (2.9% for daily vapers and 4.2% for non-daily vapers); as such, we used a random forest-based algorithm to conduct imputation [33]. Categorical variables were then converted to factor variables whereas all the numerical variables were standardized to range from 0 to 1 using minimum-maximum scalar normalization. Following this, the data were split into training and testing sets, by 80% and 20%, respectively, for both daily and non-daily vapers. For each group, a penalized regression model was first developed on the training set (see below), and then evaluated on the testing set.

### 2.6. Penalized Lasso Regression

Penalization is a technique for developing high-performance parsimonious predictive models by selecting only a subset of key predictors from a large pool of variables [27]. This is carried out by adding a penalty term to the total sum of squared errors (i.e., the loss function of the standard ordinary least-square regression) so the model is penalized for having an increased number of predictors. The parameter attached to the penalty term, denoted as lambda, controls how much coefficients of predictors shrink to 0 (i.e., severity of penalty). The optimal value of lambda is selected as the one minimizing the cross-validated prediction error rate.

In this analysis, we used the least absolute shrinkage and selection operator (lasso) penalization approach. When compared to other approaches (ridge or elastic net), lasso selects only one variable and ignores the other when highly correlated variables are present to achieve a sparse model [34]. On the training set, we identified the optimal lambda via 10-fold cross-validation where all values from 0.001 to 1 with an 0.0005 increment were searched [35].

The performance of the lasso regression model was evaluated based on the *R*-squared and the root mean-squared-error (*RMSE*) on the testing set. *R*-squared specifies how much of the variance in the vaping dependence score can be explained by the model, while *RMSE* represents how spread out (variance) the residuals are. *R*-squared is calculated as follows:

$$R^{2}=1-\frac{RSS}{TSS}$$

where *RSS* is sum of squares of residuals and *TSS* is total sum of squares. On the other hand, *RMSE* is calculated as follows:

$$RMSE=\sqrt{\sum_{i=1}^{n}\frac{{\left(\hat{y_{i}}-y_{i}\right)}^{2}}{n}}$$

where $\hat{y_{1}},\;\hat{y_{2}},\;\hat{y_{3}}$… are predicted values, $y_{1},\;y_{2},\;y_{3}$… are observed values and n is the number of observations. A model’s performance is considered high when the *R*-squared value is high and *RMSE* score is low. On the premise that our dataset contains at least some significant predictors of the vaping dependence score, an *R*-squared of 0.1 (or 10%) or above was considered acceptable [36].

### 2.7. Identifying the Top Predictors of Increased Vaping Dependence Score

Using the final model, we ranked the absolute value of each non-zero coefficient to identify the top predictors of the vaping dependence score. This is because in the lasso regression, big coefficients are associated with larger effects. To quantify the relative effect sizes of the predictors, we computed a score (0–100%) for each predictor to represent how its coefficient compared to that of the most important predictor (100%) [37]. We categorized the identified top predictors into three categories: ‘Psychological or behavioural’, ‘Socioeconomic and health status’, and ‘Commercial’. We color-coded each identified top predictor on the importance ranking plot based on the category they belonged to. We then generated separate plots for non-daily and daily vapers, displaying the combined variable importance for each category. Following this, partial dependence plots were generated for the top 20 predictors to illustrate their marginal effect on the predicted vaping dependence score [8,38,39,40]. Analyses were conducted using R version 4.2.1 (R Foundation for Statistical Computing, Vienna, Austria).

## 3. Results

### 3.1. Baseline Characteristics

Table 1 presents the characteristics of our cohort at the baseline, stratified by status of daily vaping (*n* = 1172). The mean age of the cohort was 19.6 (standard deviation [SD], 2.6) years with the majority being female (*n* = 895, 76.4%) or identifying as a woman (*n* = 762, 65.0%), White (*n* = 823, 70.2%), and single/never married (*n* = 958, 81.7%). Nearly half of all participants were from the province of Ontario (*n* = 560, 47.8%) and more than half of them were heterosexual (*n* = 601, 53.7%). At the baseline, there were slightly more daily vapers (*n* = 643, 54.9%) than non-daily vapers (*n* = 529, 45.1%) in our cohort. When compared to their non-daily vaping counterparts, daily vapers were less likely to be female (73.4% vs. 80.0%, *p* = 0.03), although their gender distribution was not statistically different (*p* = 0.37); they were also more likely to have graduated high school (57.2% vs. 49.0%, *p* = 0.02), White (73.4% vs. 66.4%, *p* = 0.01), and reside outside of Ontario (59.3% vs. 43.7%, *p* < 0.01).

### 3.2. Vaping Dependence Score over 3 Months

**Figure 1:**
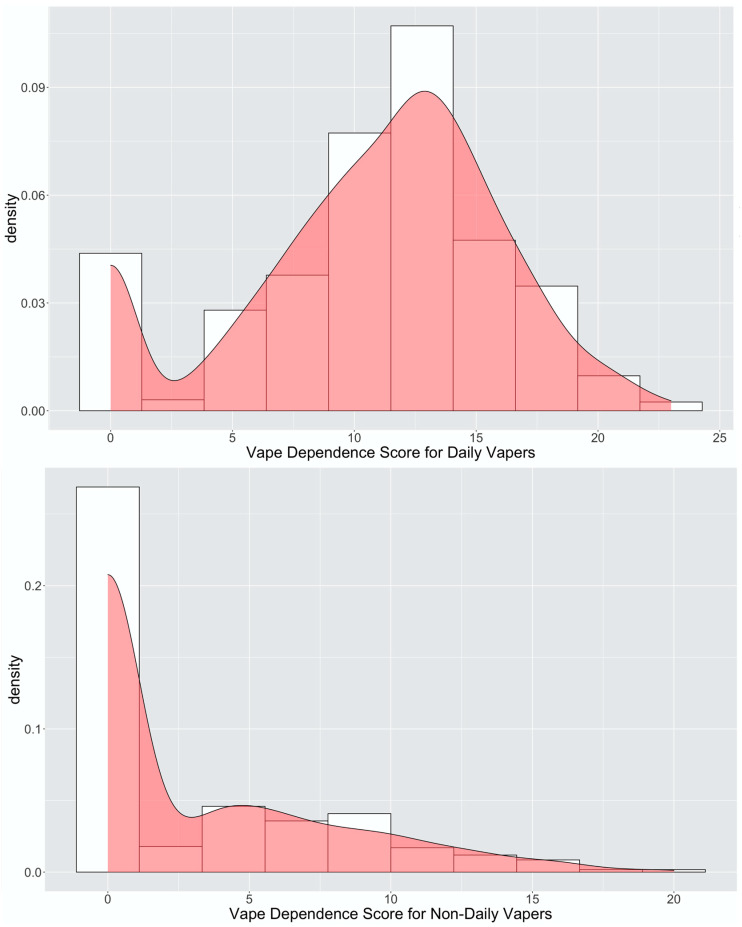
Distribution of the 3-month vaping dependence score among daily and non-daily vapers.

We illustrate the distribution of the vaping dependence score among baseline daily vapers and non-daily vapers (Figure 1). Among daily vapers, the mean score over the next 3 months was 10.72 (SD, 5.34) with median at 12 (interquartile range [IQR], 8–14). Among non-daily vapers, their 3-month vaping dependence scores were comparably lower with a right-skewed distribution with mean at 3.12 (SD, 4.50) and median at 0 (IQR, 0–6).

### 3.3. Performance of the Lasso Regression Models

Two lasso regression models were developed to predict the vaping dependence score in daily vapers and non-daily vapers. For daily vapers, the final model achieved an *R*-squared of 0.19 and an *RMSE* of 4.87 on the test set. For non-daily vapers, the model reached an *R*-squared of 0.29 and an *RMSE* 3.96 on the test set. Using the 10% threshold for the *R*-squared, both models were considered to have an acceptable performance.

### 3.4. Top Predictors of Vaping Dependence

**Figure 2:**
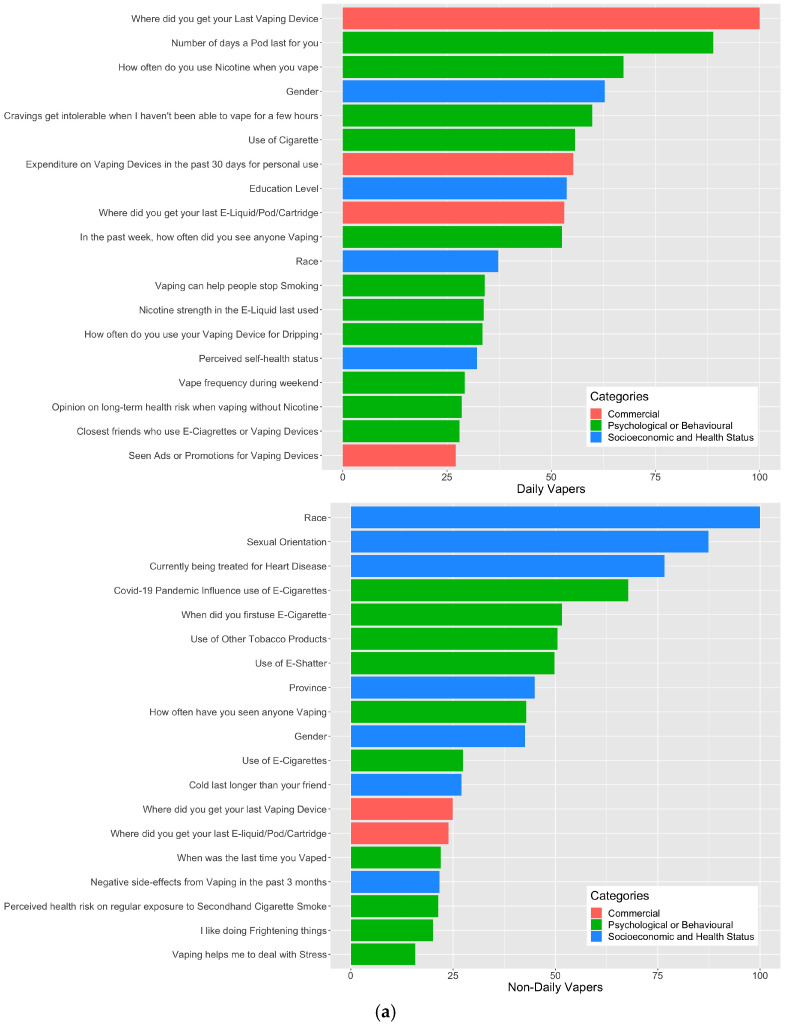

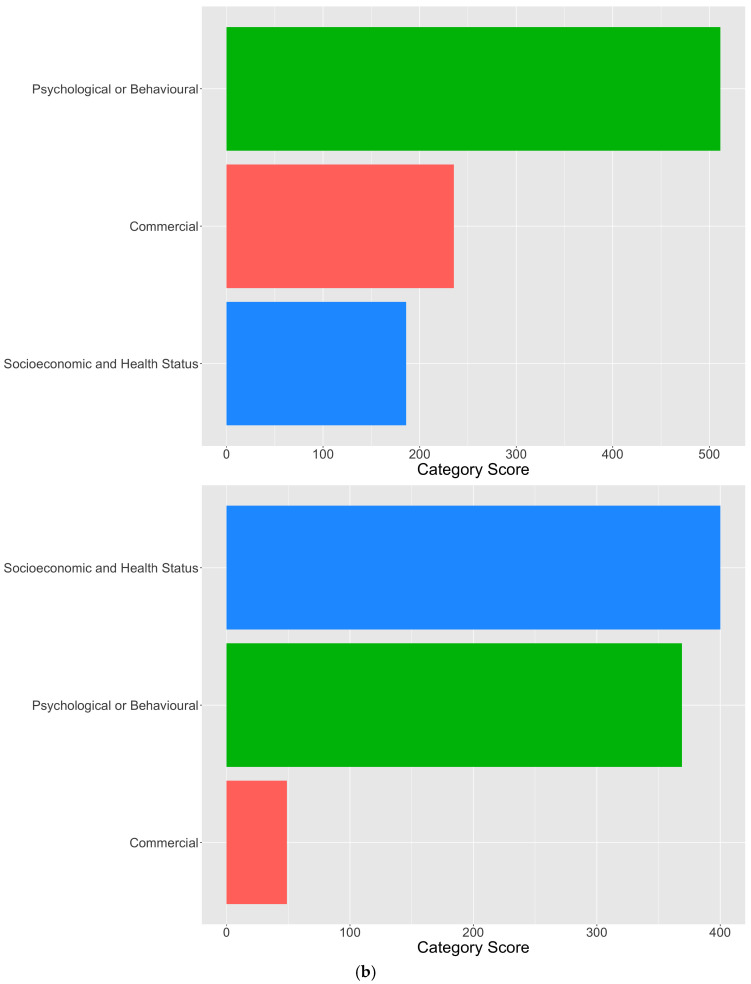
(**a**) Ranking the importance of individual predictors for 3-month vaping dependence for baseline daily (upper) and non-daily (lower) vapers. (**b**) Ranking the collective importance of “Psychological or behavioural”, “Socioeconomic and health status” and “Commercial” variables in predicting 3-month vaping dependence for baseline daily (upper) and non-daily (lower) vapers.

Using the respective lasso regression model, we identified the top 20 predictors of vaping dependence in daily vapers and non-daily vapers (Figure 2a). In general, we observed pronounced differences in the set of predictors between the two groups. Specifically, while the top predictors among daily vapers comprised largely vaping preference and behavior items (place of last vaping purchase, lasting days for a pod, and the frequency of nicotine-containing vaping), the corresponding predictors among non-daily vapers pertained to sociodemographic and health characteristics, particularly race, sexual orientation, and status of being treated for heart disease. Moreover, the pandemic impact on vaping was identified as a top predictor of 3-month vaping dependence among non-daily vapers only.

We mapped these identified predictors to three categories, including “Psychological or behavioural”, “Socioeconomic and health status” and “Commercial”, and created separate plots for non-daily and daily vapers to show the combined variable importance score for the above categories (Figure 2b). For non-daily vapers, our analysis suggested that variables falling under “Socioeconomic and health status” have the greatest combined impact on 3-month vaping dependence score, followed by variables that were “Psychological or behavioural”, and lastly, those that were of a “Commercial” nature. For daily vapers, the most important variables were “Psychological or behavioural”, followed by “Commercial” variables, and lastly, “Socioeconomic and health status” variables.

**Figure 3:**
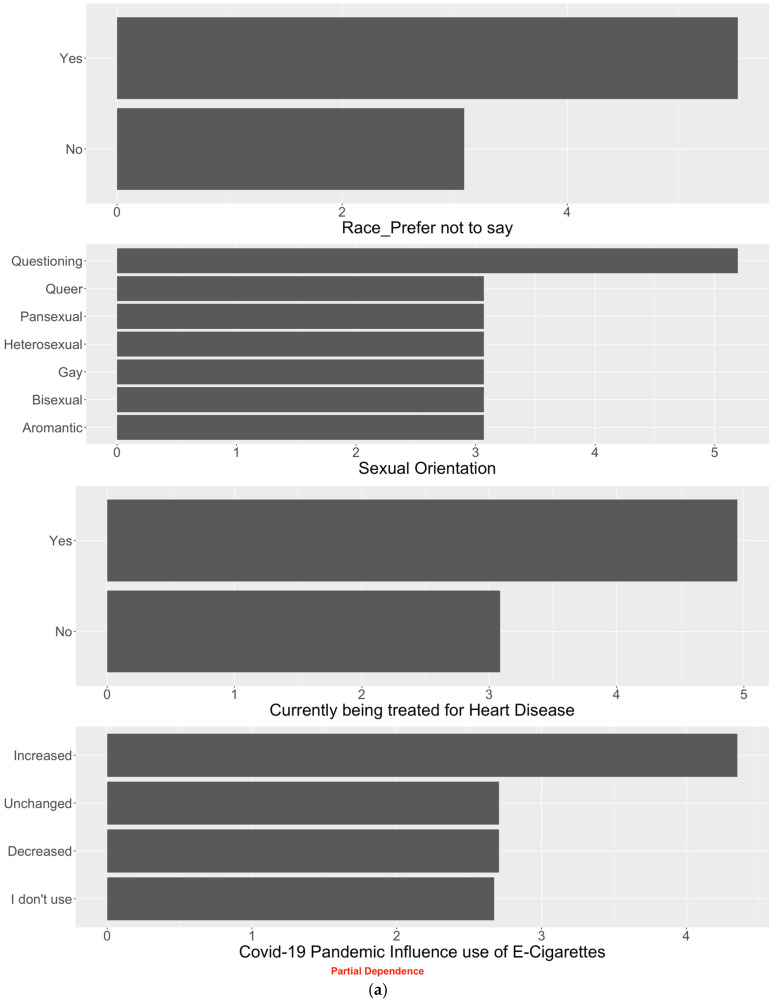

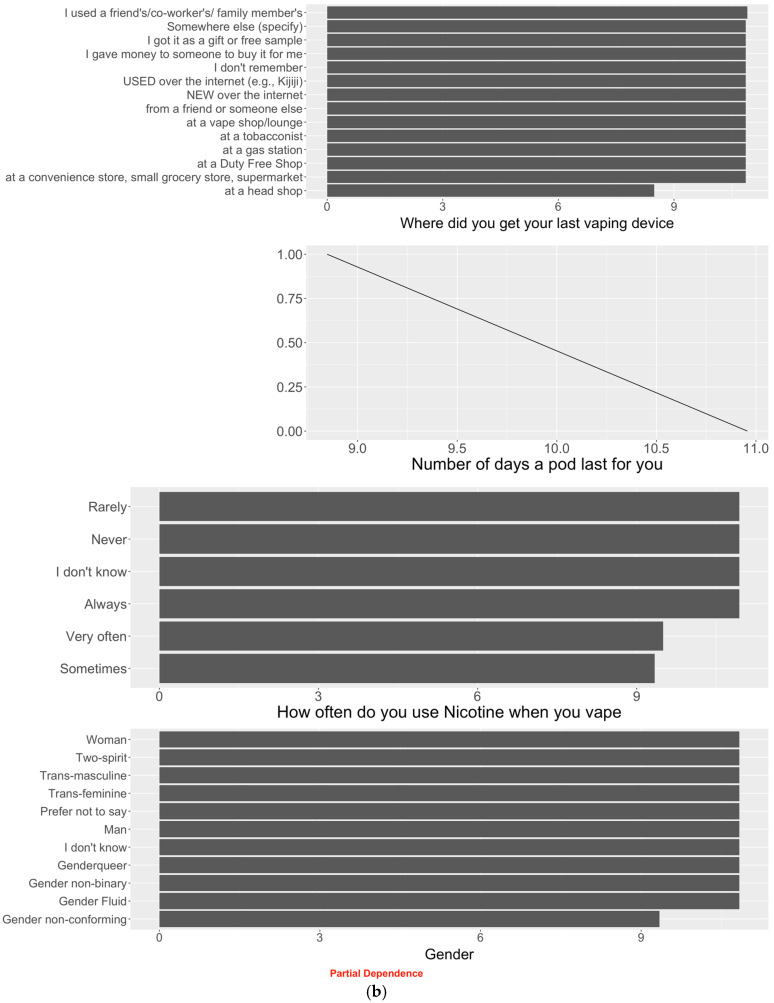
(**a**) Partial dependence plots showing the marginal effect of the identified predictors on vaping dependence score for baseline non-daily vapers. (**b**) Partial dependence plots showing the marginal effect of the identified predictors on vaping dependence score for baseline daily vapers.

We generated partial dependence plots to demonstrate the specific marginal effect of each of the identified top predictor on the predicted vaping dependence score. We presented the plots for the top 4 predictors in Figure 3a for non-daily vapers and in Figure 3b for daily vapers and reserved the remainder in Appendix B. For daily vapers, their vaping dependence score over the next 3 months decreased if they reported getting their last vaping device from a head shop (rather than elsewhere such as from the internet), using nicotine in vape ‘very often’ or ‘sometimes’, or identifying as gender non-conforming. We also observed an inverse linear association between vaping dependence score and the lasting duration of a vaping pod. Among non-daily vapers, their vaping dependence score in 3 months increased for those who did not disclose their race, had questioning sexual orientation, were currently receiving treatment for heart disease, and reported to have increased use of vaping during the COVID-19 pandemic.

## 4. Discussion

This machine learning study identified individual-level predictors of vaping dependence observed in 3 months, a score between 0–23, among daily vapers and non-daily vapers in a Canadian adolescent cohort. Our study suggests that the key predictors of vaping dependence are different among daily and non-daily vapers.

Findings of this study are unique in the international literature due to our study design, inclusion of novel variables, and use of supervised machine learning. Specifically, when compared to the existing literature, we included a broader range of participants, namely both non-daily and daily vaping adolescents from across Canada. This enhanced the generalizability of our findings which we believe represents an advancement of previous studies that tended to focus on more specific adolescent subpopulations, such as ever-vaping high school seniors from California [8]. Moreover, there were three groups of variables we assessed as candidate predictors of vaping dependence in this study that were either completely novel in the literature or were overlooked in the past. This includes (1) COVID-19 related variables such as the perceived impact of the pandemic on access to vaping products and changes in vaping frequency; (2) variables pertaining to heated tobacco products, which we operationalized as the awareness about heated tobacco products, types of products being used, and how often participants reported to use them; and (3) variables related to the promotion and advertisements of vaping, including where individuals were exposed, perceived impact on attitudes and beliefs towards vaping, and what kinds of promotions were seen. The inclusion of these variables improved the timeliness of our findings specifically in the context of building capacity for vaping prevention strategies in the post-pandemic era. Finally, we employed lasso regression, a supervised machine learning method that effectively selects the most important features by shrinking the coefficient of less important variables to zero, giving rise to a sparse model that is less prone to overfitting. The lasso model is also highly interpretable when compared to more black-box algorithms (such as the random forests [8,21]), which is critical for deriving policy-relevant findings that are accessible to stakeholders. The robustness and interpretability of the modeling results are further enhanced by our use of a random forest-based method to handle the missing data.

Among daily vapers, we found those buying from a headshop to have the lowest 3-month vaping dependence score. This might reflect the distinct characteristics of these buyers that are also commonly associated with a lower risk of continued substance use, such as older age, higher education, and having less sensation-seeking tendency [6]. Furthermore, since the purchasing of vaping products has shifted significantly from a specialty store (a vape or tobacco shops) to online over the past few years [41,42], our findings imply that due to the convenience of e-commerce, young people are indeed buying these products more frequently and ultimately showing signs of dependence. Moreover, because both of our surveys were conducted during the COVID-19 lockdown period, even more people, especially those who were somewhat dependent on vaping already, might have preferred buying their products online due to social distancing policies, the closure of vape shops, and the influence of online marketing [43]. Previous literature suggests that exposure to the marketing of tobacco products increases young people’s exposure to misleading information about the safety and effectiveness of these products, which may subsequently increase their uptake [44,45]. Additionally, the anonymity and lack of regulation associated with online sales make it easier for minors to access e-cigarettes and for vendors to engage in deceptive marketing practices. This calls for limitation, even prohibition, of vaping e-commerce to prevent an uprising of vaping dependence among young people in the aftermath of COVID-19 [42,43,46]. For example, stringent policies must be enforced to forbid online vendors to sell vaping products to underage individuals.

Additionally, for daily vapers, we found their 3-month vaping dependence to be inversely associated with the number of days their pod lasted. This is unsurprising since those who empty a pod quickly have a higher puff number and generally vape more frequently, which in turn heightens their risk of dependence. As such, the time a vape pod lasts may be a valuable quantitative indicator of consumption, even dependence. Our study also provides further evidence on the association between vaping dependence and nicotine use, including frequency and concentration [6,8,47,48,49]. Specifically, we found daily vapers who reported using nicotine ‘very often’ or ‘sometimes’, i.e., those with moderate to moderately high use of nicotine in their vape, to have the greatest tendency of showing dependence in 3 months. It is worth noting that people who reported ‘always’ using nicotine in their vape were not identified by us to be the ones with the highest 3-month vaping dependence. This may be attributed to participants not being truthful when completing the survey, a common phenomenon seen in youth and young adults [50]. Moreover, our findings can also be viewed in the light of poly-/solo-product use: those who ‘sometimes’ vaped nicotine may be using other nicotine-containing products simultaneously, resulting in their high overall dependence, whereas those who ‘always’ used nicotine in their vape may rely on vaping as the sole means to consume nicotine [51]. Nevertheless, these results call for a regulatory consensus on how nicotine strength should be labelled on vaping products and enforcement of such standards.

For non-daily vapers, we observed those who did not disclose their race had higher vaping dependence in 3 months. It is worth mentioning that only 0.4% of our cohort did not disclose their race; as such, further studies are required to confirm the robustness of this finding. Still, these results are consistent with prior studies that demonstrated high perceived discrimination by youth to be associated with not only increased uptake of vaping but more frequent use as well [8,52]. Policies promoting cultural diversity, social inclusion, and integration coupled with incident recording and monitoring might mitigate discrimination and its consequences among adolescents.

We found 3-month vaping dependence to be higher for non-daily vapers reporting a questioning sexual orientation. This is consistent with a multitude of statistics documenting the high prevalence of substance use among sexual/gender minority individuals; notably, prevalence of current vaping in this population has been estimated to be twice that of heterosexuals [1,53,54]. However, there is no data on the particular association between substance abuse and having a questioning sexual orientation. As such, our analysis helped fill this literature gap by suggesting that young people who are in the process of figuring out their sexual orientation may be at exceedingly high risk of using vaping as a coping mechanism, a harmful decision for their health [39,40]. We also reached similar findings among baseline daily vapers as being gender non-conforming was identified to be one of the most important predictors of vaping dependence in this cohort. Jointly, these results illustrate the importance of considering sexual/gender orientations in the planning of youth vaping prevention programs. Concerted efforts are needed to educate the sexual/gender minorities (especially those with a questioning sexual orientation and/or gender non-conforming identity) about the potential harms of vaping. At the very least, they must be made aware of better coping mechanisms to deal with stress and anxiety faced during the questioning phase of life.

### Limitations

This study is not without limitations. Firstly, as we relied on social media platform (Instagram) to recruit participants, this may have limited the representativeness of the study cohort as social media users were associated with unique characteristics. Additionally, both surveys were administered online, raising concerns on recall bias as adolescents are known to present themselves in a more favorable light or conform to social expectations and norms when completing such online surveys, particularly those related to substance use and mental health. Non-response bias was also a concern; however, we utilized a rigorous random forest-based algorithm to impute the infrequent missing data and believe that we have mitigated this issue. Still, researchers with access to health administrative data should consider linking them with survey responses to form a more reliable dataset. Next, despite the satisfactory performance of our two models, the sample size of this study was not substantial. As this longitudinal survey study is still ongoing and we are recruiting more adolescents, we plan to apply a time-to-event analytical framework once more waves of data become available using either machine learning (such as random survival forests) or non-machine learning techniques (such as Cox regression) to assess the trajectory and time-dependent predictors of vaping dependence. To preserve the original questionnaire design, we did not conduct more extensive data pre-processing procedures, and instead, entered all the variable levels directly into model training. This might have led to potential biases and a reduced model performance due to the presence of imbalanced variables [55]. However, the primary goal of this study was not to devise a predictive model, but to explore meaningful predictors of vaping dependence by screening a large pool of variables. As such, we recommend future predictive modelling studies to incorporate more in-depth data pre-processing procedures to achieve a powerful and robust predictive model. Additionally, although the vaping dependence score we used had been validated on a Canadian youth cohort [5], its reliability when applied to a non-Canadian and/or older population is unknown. Lastly, we were unable to draw any causal conclusions, although lasso regression does yield some implications for causality [56]. Future research with a hypothesis-testing design should build upon our results to establish whether the identified predictors constitute true causes of vaping dependence.

## 5. Conclusions

We followed a group of ever-vaping adolescents from across Canada for 3 months to observe their self-reported vaping dependence. By using machine learning, we identified different predictors of vaping dependence for baseline daily and non-daily vapers. The resulting models performed moderately well, with an *R*-squared of 0.19 and an *RMSE* of 4.87 for daily vapers, and an *R*-squared of 0.29 and an *RMSE* of 3.96 for non-daily vapers. The top predictors of vaping dependence identified for daily vapers were the place of last vaping purchase, number of days a pod lasted, and the frequency of nicotine-containing vaping. In contrast, the top predictors for non-daily vapers were race, sexual orientation, and being treated for heart disease. Overall, we found psychological/behavioral factors to influence vaping dependence the most for daily vapers, while socioeconomic and health variables tended to affect non-daily vapers the most. For non-daily vapers only, those who reported to have increased vaping during the first peak of the COVID-19 pandemic had a higher dependence score in 3 months. Overall, these findings show factors escalating short-term dependence to be different among adolescents who have already formed a daily habit of vaping and those who are currently vaping less frequently. This means interventions aimed at reducing vaping among adolescents need to adopt a personalized approach and, at the very least, consider the current vaping frequency of the targeted adolescents. Future studies should confirm the causal role of the identified predictors to enhance evidence-based policy formulation.

## Figures and Tables

**Table 1:** Baseline sociodemographic characteristics of ever-vaping adolescents recruited from across Canada stratified by baseline daily vaping status (*n* = 1172).

Variables	Daily Vapers (*n* = 643, 54.9%)	Non-Daily Vapers (*n* = 529, 45.1%)	Total (*n* = 1172)	*p*-Value
Age, year, mean (SD)	19.6 (2.5)	19.6 (2.7)	19.6 (2.6)	0.94
**Sex**				0.03
Female	472 (73.4)	423 (80.0)	895 (76.4)	
Male	163 (25.3)	102 (19.3)	265 (22.6)	
Prefer not to say	8 (1.2)	4 (0.8)	12 (1.0)	
**Education**				0.02
Below high school	135 (21.0)	128 (24.2)	263 (22.4)	
Completed high school	368 (57.2)	259 (49.0)	627 (53.5)	
College diploma	63 (9.8)	52 (9.8)	115 (9.8)	
University degree or above	77 (12.0)	90 (17.0)	167 (14.2)	
**Race**				0.01
White	472 (73.4)	351 (66.4)	823 (70.2)	
Indigenous	54 (8.4)	35 (6.6)	89 (7.6)	
Chinese	23 (3.6)	31 (5.9)	54 (4.6)	
South Asian	25 (3.9)	26 (4.9)	51 (4.4)	
Southeast Asian	4 (0.6)	5 (0.9)	9 (0.8)	
Filipino	8 (1.2)	9 (1.7)	17 (1.5)	
Japanese/Korean	8 (1.2)	9 (1.7)	17 (1.5)	
West Asian	13 (2.0)	26 (4.9)	39 (3.3)	
Latin American	14 (2.2)	20 (3.8)	34 (2.9)	
Black	13 (2.0)	15 (2.8)	28 (2.4)	
Others	9 (1.4)	2 (0.4)	11 (0.9)	
**Province**				<0.01
Ontario	262 (40.7)	298 (56.3)	560 (47.8)	
Alberta	134 (20.8)	82 (15.5)	216 (18.4)	
British Columbia	110 (17.1)	77 (14.6)	187 (16.0)	
Quebec	27 (4.2)	18 (3.4)	45 (3.8)	
Saskatchewan	35 (5.4)	8 (1.5)	43 (3.7)	
Manitoba	25 (3.9)	18 (3.4)	43 (3.7)	
Nova scotia	17 (2.6)	21 (4.0)	38 (3.2)	
New Brunswick	15 (2.3)	2 (0.4)	17 (1.5)	
Newfoundland and Labrador	10 (1.6)	4 (0.8)	14 (1.2)	
Prince Edward Island	7 (1.1)	1 (0.2)	8 (0.7)	
Northwest territories	1 (0.2)	0 (0.0)	1 (0.1)	
**Marital status**				0.21
Single or never married	517 (80.4)	441 (83.4)	958 (81.7)	
Married or living with a partner	126 (19.6)	87 (16.4)	213 (18.2)	
Divorced, separated, or widowed	0 (0.0)	1 (0.2)	1 (0.1)	
**Sexual orientation**				0.74
Heterosexual	340 (55.1)	261 (52.0)	601 (53.7)	
Bisexual	195 (31.6)	176 (35.1)	371 (33.2)	
Pansexual	33 (5.3)	28 (5.6)	61 (5.5)	
Gay	26 (4.2)	15 (3.0)	41 (3.7)	
Queer	17 (2.8)	16 (3.2)	33 (2.9)	
Aromantic	4 (0.6)	4 (0.8)	8 (0.7)	
Questioning	1 (0.2)	2 (0.4)	3 (0.3)	
Demisexual	1 (0.2)	0 (0.0)	1 (0.1)	
Missing	26 (4.0)	27 (5.1)	53 (4.5)	
**Gender**				0.37
Woman	402 (62.5)	360 (68.0)	762 (65.0)	
Man	146 (22.7)	91 (17.2)	237 (20.2)	
Gender non-binary	34 (5.3)	27 (5.1)	61 (5.2)	
Transgender	20 (3.1)	18 (3.4)	38 (3.2)	
Gender fluid	9 (1.5)	5 (1.0)	14 (1.2)	
Two-spirit	3 (0.5)	2 (0.4)	5 (0.4)	
Gender non-conforming	2 (0.3)	1 (0.2)	3 (0.3)	
Gender queer	1 (0.2)	2 (0.4)	3 (0.3)	
Prefer not to say	26 (4.0)	23 (4.3)	49 (4.2)	

Notes: Unless otherwise specified, we reported the count and percentage in each cell. Pearson’s Chi-Square tests and *t*-tests were used to examine the distribution of categorical and continuous variables by status of baseline daily vaping.

**Figure A2:**
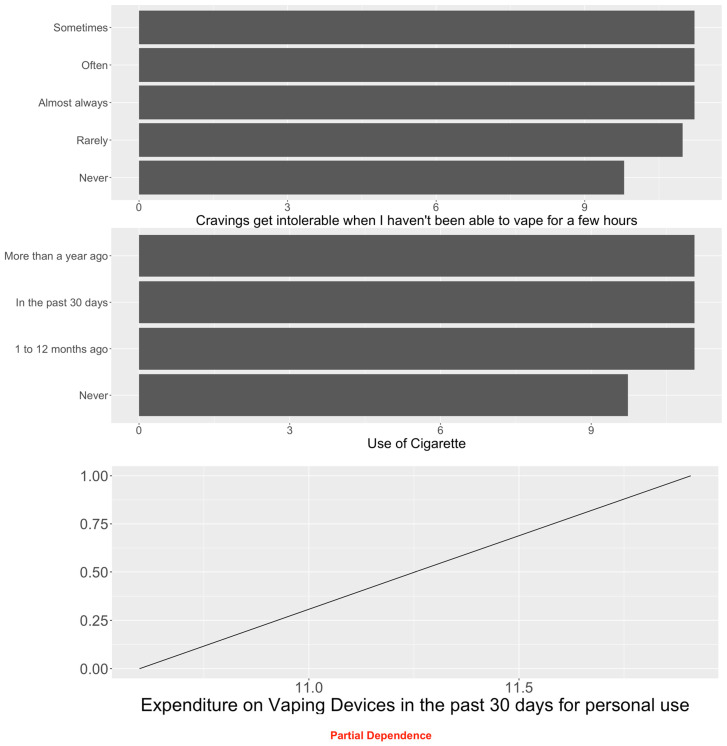
Partial dependence plots among daily vapers: frequency of cravings getting intolerable when I have not been able to vape for a few hours, time of last cigarette use, expenditure on vaping devices in the past 30 days for personal use.

**Figure A3:**
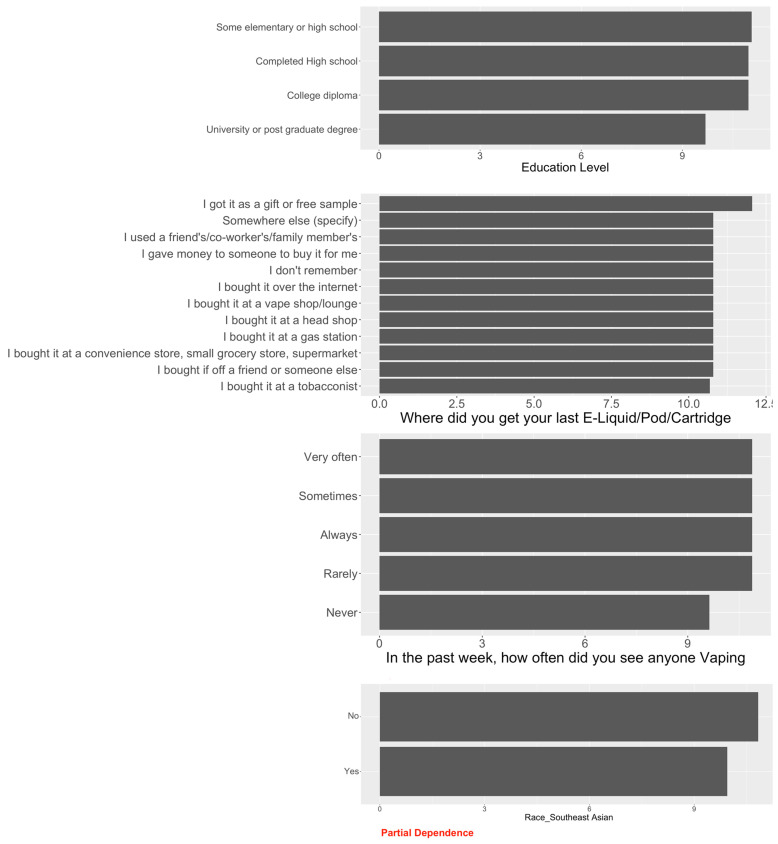
Partial dependence plots among daily vapers: education, place of acquisition/buying of last e-liquid/pod/cartridge, frequency of seeing anyone v aping in the past week, and race.

**Figure A4:**
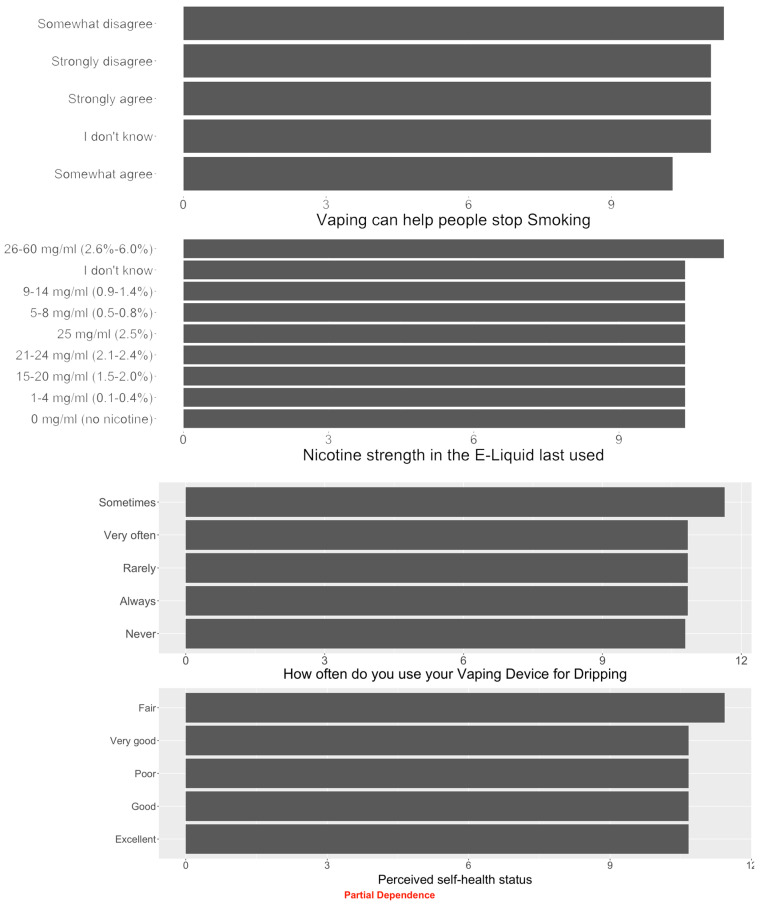
Partial dependence plots among daily vapers: Degree of concurrence with the proposition that ‘Vaping can help people stop smoking’, nicotine strength in the e-liquid last used, frequency of use of vaping device for dripping, and perceived self-health status.

**Figure A5:**
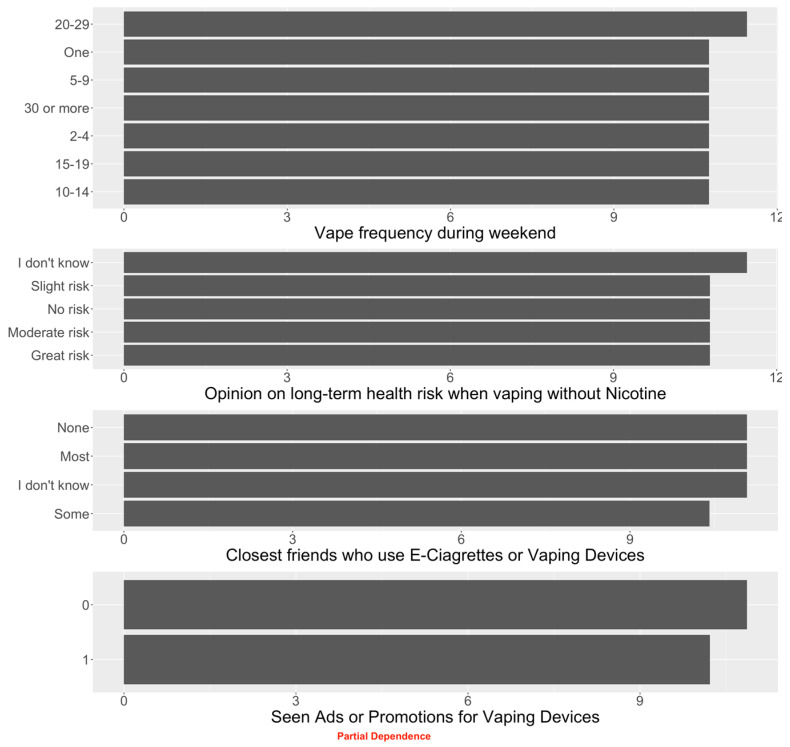
Partial dependence plots among daily vapers: Frequency of vaping during weekend, opinion on long-term health risk when vaping without nicotine, closest friends who use e-cigarettes or vaping devices, influence of seeing ads or promotions for vaping devices.

**Figure A6:**
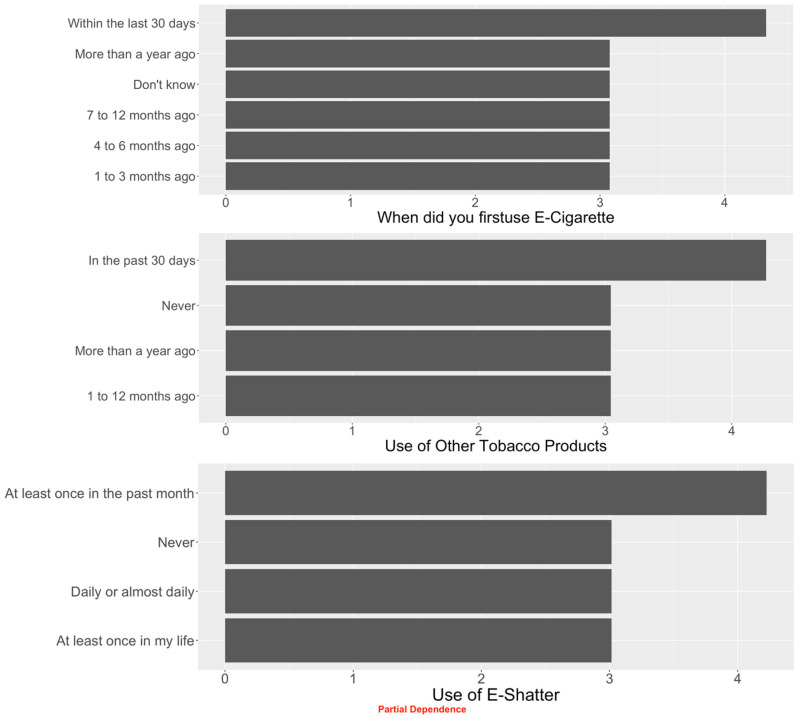
Partial dependence plots among non-daily vapers: use of first e-cigarette, last use of other tobacco products, last use of e-shatter.

**Figure A7:**
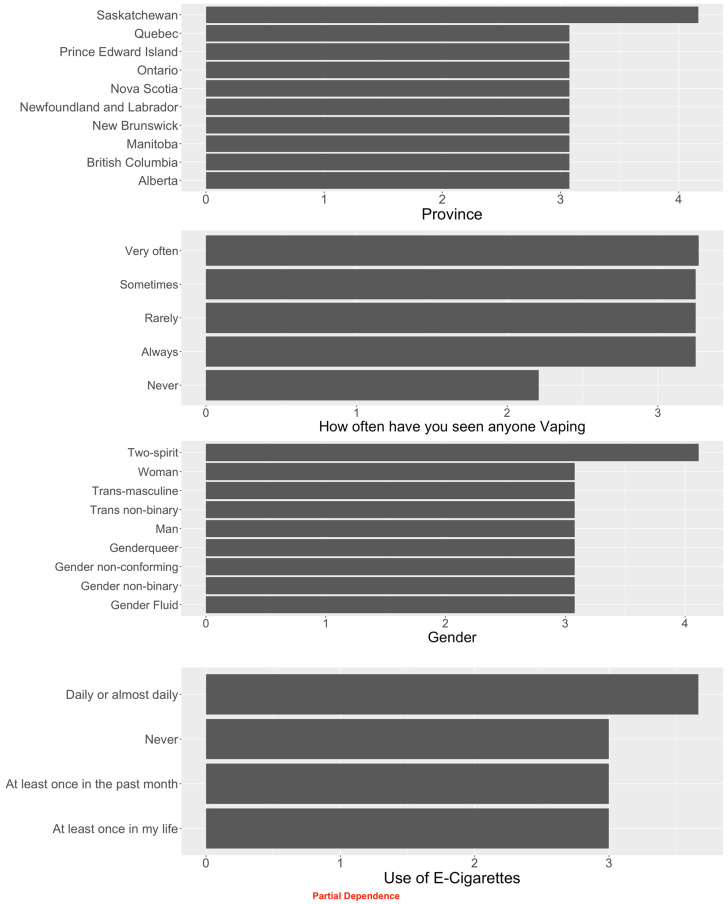
Partial dependence plots among non-daily vapers: province, frequency of seeing anyone vaping, gender, last use of e-cigarettes.

**Figure A8:**
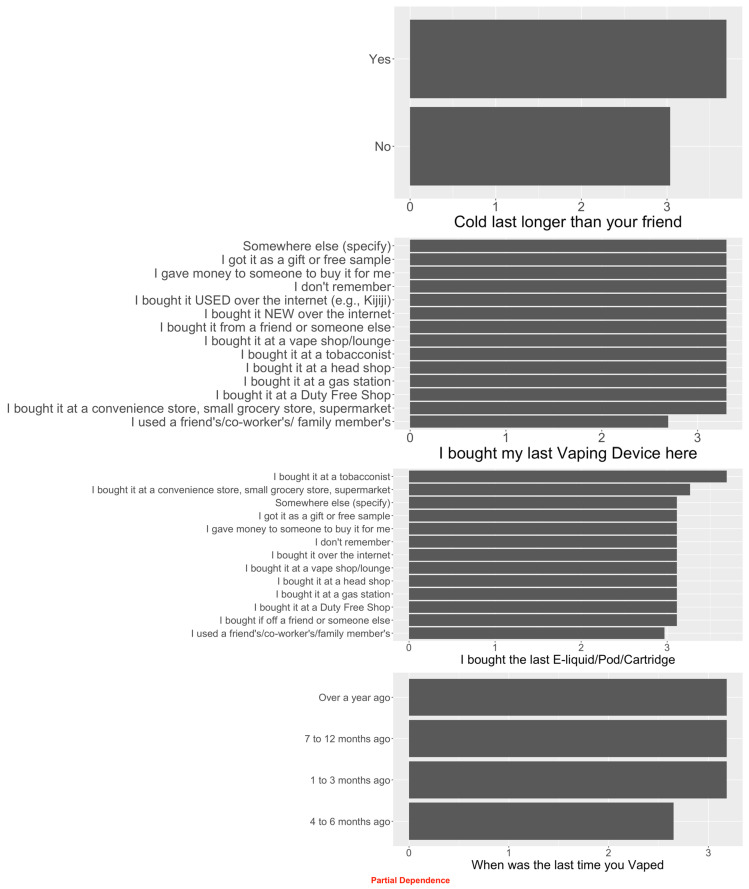
Partial dependence plots among non-daily: exhibition of extended duration of cold when compared to peers, place of purchase of last vaping device, place of purchase of last e-liquid/pod/cartridge, last use of vape.

**Figure A9:**
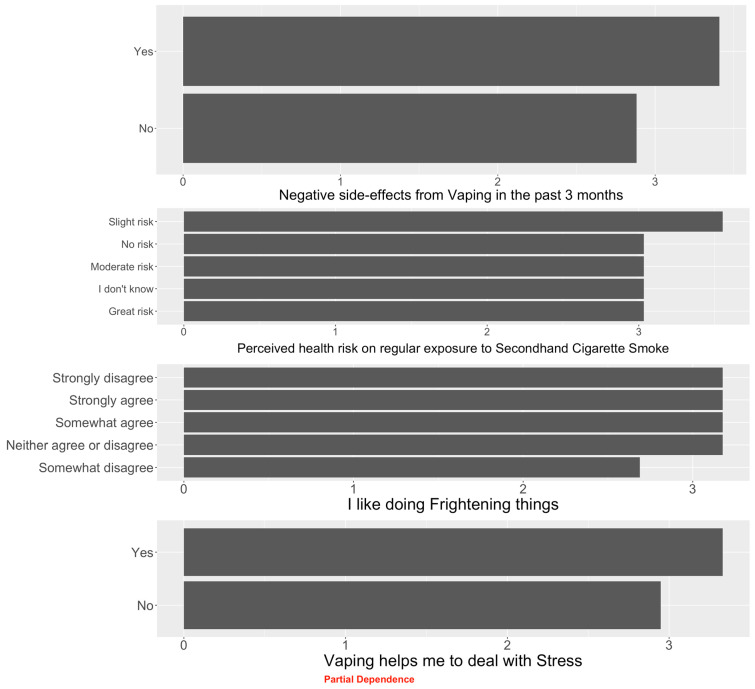
Partial dependence plots among non-daily vapers: exhibition of negative side-effects from vaping in the past 3 months, perceived health risk on regular exposure to secondhand cigarette smoke, degree of concurrence with the proposition that ‘I like doing frightening things’, perception of individuals on the statement ‘Vaping helps me deal with stress’.

## Data Availability

The R codes used in this analysis are available on the Open Science Framework: https://osf.io/czs9e/. Person-level survey data used in this analysis are not publicly available. Request to access these data can be made by contacting the corresponding author, Michael Chaiton, at michael.chaiton@camh.ca.

## Appendix A

**Table A1:** Calculation of the vaping dependence score at the 3-month follow-up.

Survey Questions	Question Responses and Scores Allocated
On days that you can vape freely, how soon after you wake up do you have the first vape of the day?	More than 120 min = 1, 61–120 min = 2, 31–60 min = 3, 16–30 min = 4, 6–15 min = 5, 0–5 min = 6
When you vape, how many puffs do you take?	Less than 5 = 1, 5–9 = 2, 10–29 = 3, 30 or more = 4;
Do you sometimes awaken at night to vape?	Yes = 1, No = 0
How many nights per week do you typically awaken to vape?	One Night = 1, Two or Three Nights = 2, Four or More Nights = 3
Do you ever have strong cravings to vape?	Yes = 1, No = 0
Over the past week, how strong have the urges to vape been?	None = 1, Slight = 2, Moderate = 3, Strong = 4, Very Strong = 5
Is it hard to keep from vaping in places where you are not supposed to?	Yes = 1, No = 0
Did you feel more irritable because you could not vape?	Yes = 1, No = 0
Did you feel nervous, restless, or anxious because you could not vape?	Yes = 1, No = 0

Notes: In the analysis, we excluded participants who did not answer any of the 9 questions from the cohort. For those who answered only some of the nine questions, a score of 0 was allotted.

## Appendix B

We present the one-way partial dependence plot of each identified top predictors of 3-month vape dependence score for baseline daily and non-daily vapers. Variable values were plotted on the x-axis while the partial dependence was depicted on the y-axis.
